# Research on the comparison of impact resistance characteristics between energy absorption and conventional hydraulic columns in fluid–solid coupling

**DOI:** 10.1038/s41598-023-47887-y

**Published:** 2023-11-25

**Authors:** Jianzhuo Zhang, Hao Guo, Yonghui Xiao, Yishan Pan, Kai Xu, Chenguang Guo, Baojun Ni, Fengnian Zhao

**Affiliations:** 1 School of Mechanical Engineering, Liaoning Technical University, Fuxin, 123000 China; 2 School of Physics, Liaoning University, Shenyang, 110000 China; 3 Shandong Yankuang Intelligent Manufacturing Co., Jining, 272000 China

**Keywords:** Mechanical engineering, Fluid dynamics, Solid Earth sciences

## Abstract

Rock burst disaster affects underground mining safety. The energy-absorbing hydraulic support for preventing tunnel impact has been implemented in rock burst mines. In order to compare the impact resistance characteristics of conventional columns and energy-absorbing columns, based on the derivation of energy theory, the CEL fluid–solid coupling simulation algorithm is used to simulate the process of static load 1000 kN superimposed impact load 1500 kN on φ180 mm type conventional column and energy-absorbing column. Combined with the static-dynamic combined test of the 6500 kN impact testing machine on the column, the accuracy and reliability of the CEL simulation column impact response are verified. The results showed that compared with conventional columns, the reaction force of energy-absorbing columns is reduced by 32.55%. The stress and expansion of the cylinder are significantly reduced. The acceleration of the mass movement has been reduced by 59.46%. The addition of the energy-absorbing device enhances the system's energy absorption by 33.46%, thereby reducing the energy absorption of the column itself to 23.58%. Additionally, the deformation of the energy-absorbing device increases the effective displacement by 239.45%. This also prolongs the impact duration, ensuring sufficient time for the safety valve to open, safeguarding the support from damage, and enhancing the overall integrity of the tunnel.

## Introduction

China's well mining depth has been increasing year by year, with the average mining depth of the working face reaching around 700 m. Kilometer deep wells have also become a significant proportion^1–4^. As the mining depth increases, the stress level in the mining field gradually rises, leading to a higher possibility of rock burst. Furthermore, the mining space expands, resulting in an increase in the overlying rock layer in the mining field. This increase significantly contributes to the occurrence of power phenomena in the mining field and may trigger the linkage of mining airspace induced by rock burst^5–8^. Statistics show that approximately 90% of the total number of rock burst occurrences in coal mines in China happen in the overrun tunnel during the mining period^9–11^. Therefore, the support for overrun tunnel anti-impact has become a crucial concern in the industry.

**Figure 1:**
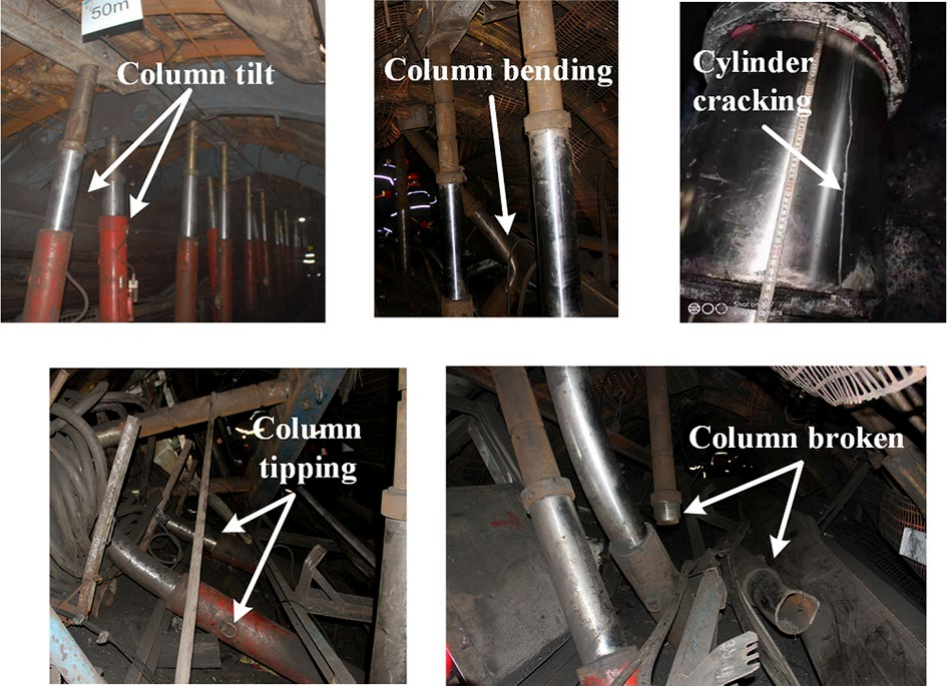
Damage to the column caused by rock burst.

The occurrence of rock burst is a process of energy conversion. Since the safety valve cannot be opened effectively in a very short time, the hydraulic support often bears dynamic loads that exceed its rated working resistance. This can lead to various issues such as cylinder expansion, bending, cracking, tipping, and column bursting, as shown in Fig. 1. When the support fails, the tunnel loses its final protective barrier, resulting in the narrowing or even closure of the tunnel. To address the hydraulic support is not enough to resist the problem of rock burst, our scholars have proposed the concept of "rigid-flexible coupling" support^12^. This involves incorporating energy-absorbing components into the support, through the plastic deformation of the energy-absorbing components to achieve the absorption of the energy released during a rock burst. In this regard, Ma^13^ designed the energy-absorbing components, combined with theoretical analysis, numerical simulation, and experimental verification to assess the feasibility of energy-absorbing components; Yang^14^ proposed the application of expanding-type energy-absorbing components in energy-absorbing anti-impact support, which improved the column's resistance to impact. Wang^15^ designed a new type of ribbed plate round tube energy-absorbing components and variable-gradient thin-wall energy-absorbing components, which demonstrated a more effective energy-absorbing capability. Zhang^16^ studied the welding performance of the cover plate of pre-folded energy-absorbing components and demonstrated that increasing the cover plate can effectively reduce the "W" effect of pre-folded energy-absorbing components. Dai^17^ on the tearing stability of axial split energy-absorbing components. Through field testing, it was proven that they have a good initial splitting capability, which allows for effective dissipation of pressure energy. At this stage, the energy-absorbing anti-impact support is being used in numerous rock burst mines and has been acknowledged by enterprises.

This study is based on the energy theory and focuses on analyzing the conventional hydraulic column liquid impact problem when the safety valve fails to open effectively during impact, using a φ180 mm type (rated working resistance of 1000 kN) hydraulic column as the research object. The CEL (Coupled Eulerian–Lagrangian) fluid–solid coupling algorithm is used to simulate the energy conversion process of impact ground pressure caused by the mass impact. Impact tests are conducted using a 6500 kN hydraulic impact testing machine and compared with the simulation for validation. The dynamic response characteristics of the energy-absorbing anti-impact column under impact conditions are simulated using the CEL fluid–solid coupling algorithm. Additionally, the anti-impact characteristics of the energy-absorbing anti-impact column are quantitatively explained based on multiple sets of indicators.

## Conventional column impact analysis

**Figure 2:**
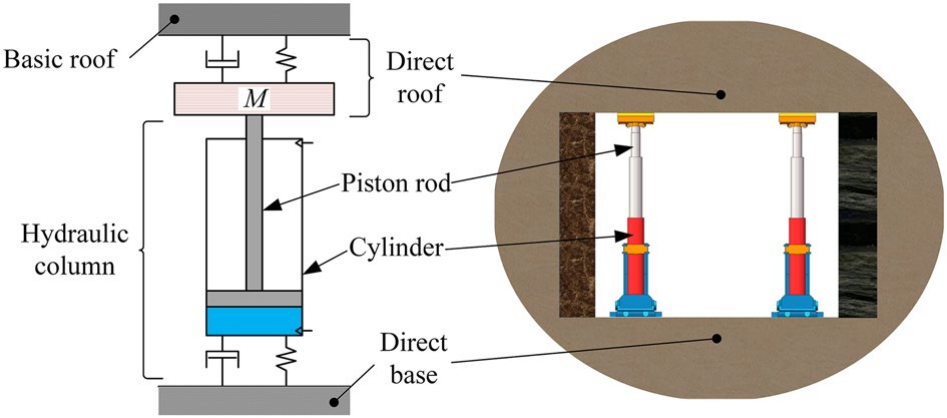
Simplified model of column load mechanics.

As shown in Fig. 2, the tunnel support is a dynamic balance system composed of a basic roof-direct roof-hydraulic support-direct base. The column bears the quasi-static load conventionally, and the energy released during a rock burst is exerted on the hydraulic column in the form of kinetic energy. Based on a simplified model of the conventional column, this study utilizes energy theory to analyze the entire system. It simplifies complex factors, establishes a cylinder-emulsion liquid series coupling system, and analyze the dynamic parameters of the column.

The inner cavity of the column is emulsion, and the following conditions are assumed for the impact calculation of this system^18^:

① The top plate of the hydraulic support is bound to the piston rod of the column, functioning as a single unit and having an equivalent mass. The hydraulic support bottom plate and column cylinder are bound together, effectively combining their qualities.
② When the direct roof and column come into contact, they remain in contact without separation, forming a unified motion system.
③ During the release of ground pressure in a rock burst, the inertia force of the hydraulic column is negligible, and all materials follow *Hooke's* law.
④ The impact process does not consider the loss of heat energy or local plastic deformation of contact parts. The test column cylinder does not experience axial movement.
⑤ Neglect the friction between the emulsion and the column body, as well as between the column body and cylinder, is neglected.

The emulsion is treated as an ideal fluid, and the gravitational force of its mass element is not counted.

1. Equivalent stiffness
  When the test column is impacted, the column cylinder body is filled with high-pressure emulsion. At this point, the emulsion and column cylinder act as a liquid spring and solid spring, respectively. The equivalent stiffness of the system can be calculated using the series theory.
  1 $$K=\frac{{K}_{y}{K}_{t}}{{K}_{y}+{K}_{t}}$$
  Here: *K*_*y*_ represents the emulsion equivalent stiffness, and *K*_*t*_ represents the cylinder equivalent stiffness.
  ① Calculation of the equivalent stiffness of emulsion
    2 $${K}_{y}=\frac{{F}_{c}}{\Delta h}=\frac{A{E}_{y}}{h}$$
    Here, *F*_*c*_ represents the varying external load, *Δh* represents the amount of change in emulsion height, *E*_*y*_ represents the modulus of elasticity of the emulsion. *A* represents the cross-sectional area of the cylinder, and *h* represents the height of the emulsion column.
  ② Calculation of cylinder equivalent stiffness
    Emulsion pressure will be equal throughout the cavity, based on the equilibrium relationship in the cylinder.
    3 $${K}_{t}={k}_{ts}A=2\delta {E}_{t}$$
    Here, *k*_*ts*_ represents the volume elasticity coefficient of the cylinder, $\delta$ is the cylinder wall thickness, and *E*_*t*_ is the elastic modulus of the cylinder. The equivalent stiffness of the column system can be calculated as follows:
    4 $$K=\frac{2\pi {r}^{2}\delta {E}_{y}{E}_{t}}{\pi {r}^{2}{E}_{y}+2\delta h{E}_{t}}$$
    Here, *r* represents the inner diameter of the cylinder, which is the cylinder inner diameter, *r* = *d*/2.
2. Dynamic load coefficient
  By applying the principle of conservation of energy, the dynamic load coefficient can be determined when an impact occurs.
  5 $${k}_{d}=1+\sqrt{1+\frac{2{W}_{E}}{Mg{\Delta }_{st}}}=1+\sqrt{1+\frac{2hk}{Mg}}$$
  Here, *W*_*E*_ represents the kinetic energy of the weight at the moment of impact contact, *M* represents the mass of the weight, ${\Delta }_{st}$ represents the displacement of the line along the direction of impact when the mass applies a static load on the column, ${\Delta }_{st}=Mg/k$. The impact force is:
  6 $${F}_{c}={k}_{d}Mg$$
3. Impact displacement
  From the characteristics of the elastomer, the dynamic load factor of the impact column is:
  7 $${k}_{d}=\frac{\Delta }{\Delta st}$$
  Here, $\Delta$ represents the descending displacement of the live column caused by the impact of the mass.
  8 $$\Delta =\frac{Mg}{k} \left(1+\sqrt{1+\frac{2hk}{Mg}} \right)$$
4. Impact time
  When a mass impacts the column, the column experiences a reaction force. This can be determined using the D'Alembert formula.
  9 $${F}_{c}-Mg-Ma=0$$
  Here, *a* represents the acceleration that is opposite to the direction of motion of the mass.
  10 $$\frac{1}{2}k{x}^{2}-Mgx+\frac{1}{2}M{v}^{2}=0$$
  Impact time can be determined.
  11 $$t={\int }_{0}^{\Delta }\frac{1}{v}dx=\sqrt{\frac{M}{k}} \left[\mathrm{arcsin}1+\mathrm{arcsin}\left(\frac{1}{{K}_{d}}-1\right)\right]$$

## Conventional column impact simulation based on CEL simulation algorithm

### CEL simulation algorithm

**Figure 3:**
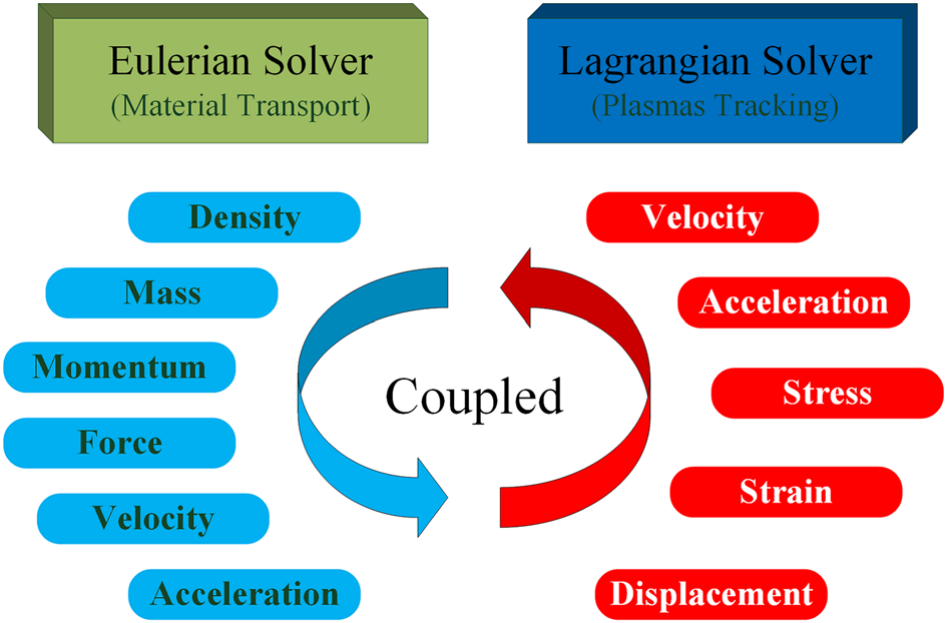
Coupled Eulerian–Lagrangian process.

The Coupled Eulerian–Lagrangian (CEL) algorithm is a fully coupled fluid–solid domain interaction algorithm. In the simulation process, the Lagrangian algorithm describes the mechanics of the structural body, while the Eulerian algorithm describes the mechanics of the fluid. These two meshes can be coupled together simultaneously for interactive computation, as shown in Fig. 3. Compared to traditional simulation studies, the CEL algorithm can effectively simulate fluid compressibility during various bearing stages. As the column is often subjected to a certain quasi-static load before being exposed to dynamic loading. Most scholars studying the impact simulation of columns often utilize the piston and cylinder binding algorithm or internal pressure mode. However, these algorithms are often inaccurate in describing the impact characteristics of hydraulic columns. The CEL algorithm can not only describe the fluid–solid coupling process but also simulate the static and dynamic load superimposed impact conditions of the column. This algorithm is more suitable for the actual working conditions of the loaded column and effectively improves the accuracy of the simulation results^19,20^.

In the standard Lagrangian description, the time derivative of the material is used, while in the Eulerian description, the time derivative of space is used. The relationship between the two is:

12 $$\frac{d\phi }{dt}=\frac{\partial \phi }{\partial t}+v\cdot (\nabla \phi )$$

Here, $\phi$ represents an arbitrary solution variable; $v$ represents the material velocity; $d\phi /dt$ represents the time derivative of the material, and $\partial \phi /\partial t$ represents the time derivative of space. The Lagrangian equations for the conservation of mass, momentum, and energy are transformed into the Eulerian conservation equations.

13 $$\left[\begin{array}{l}\frac{\partial \rho }{\partial t}+\nu \cdot (\nabla \rho )+\rho \nabla \cdot \nu =0\\ \frac{\partial \rho }{\partial t}+\nu \cdot (\nabla \cdot \nu )=\frac{1}{\rho }(\nabla \cdot \lambda )+\xi \\ \frac{\partial e}{\partial t}+\nu \cdot (\nabla e)=\lambda :\chi \end{array}\right.$$

Here, *ρ* represents density;* λ* represents the Cauchy force; *ξ* represents the body force vector; *e* represents the strain energy; and *χ* represents the velocity strain.

### CEL model preprocessing

#### Models and materials

**Figure 4:**
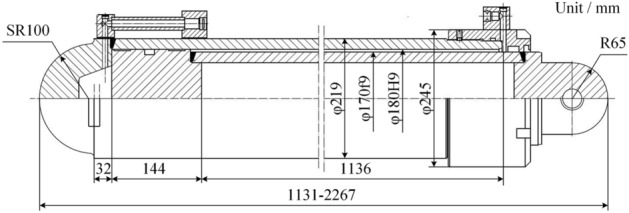
Structure dimensions of column.

The study models the φ180 mm type single telescopic column model, and its structural dimensions are shown in Fig. 4. The column structure has been simplified, and the guide sleeve is attached to the cylinder. Since the test base and the top indenter are not the objects of study, they are set to be rigid in the simulation process. The cylinder material is 27SiMn, the piston rod material is 45 steel, and the material parameters are shown in Table 1.

**Table 1:** Material parameter table.

Project	Piston rod	Cylinder
Material	45^#^Steel	27SiMn
Modulus of elasticity *E* (GPa)	209	206
Poisson's ratio *μ*	0.269	0.3
Density *ρ* (kg/m^3^)	7890	7800
Yield strength *σ*_*b*_ (MPa)	355	835
Tensile strength *σ*_*s*_ (MPa)	600	980

**Figure 5:**
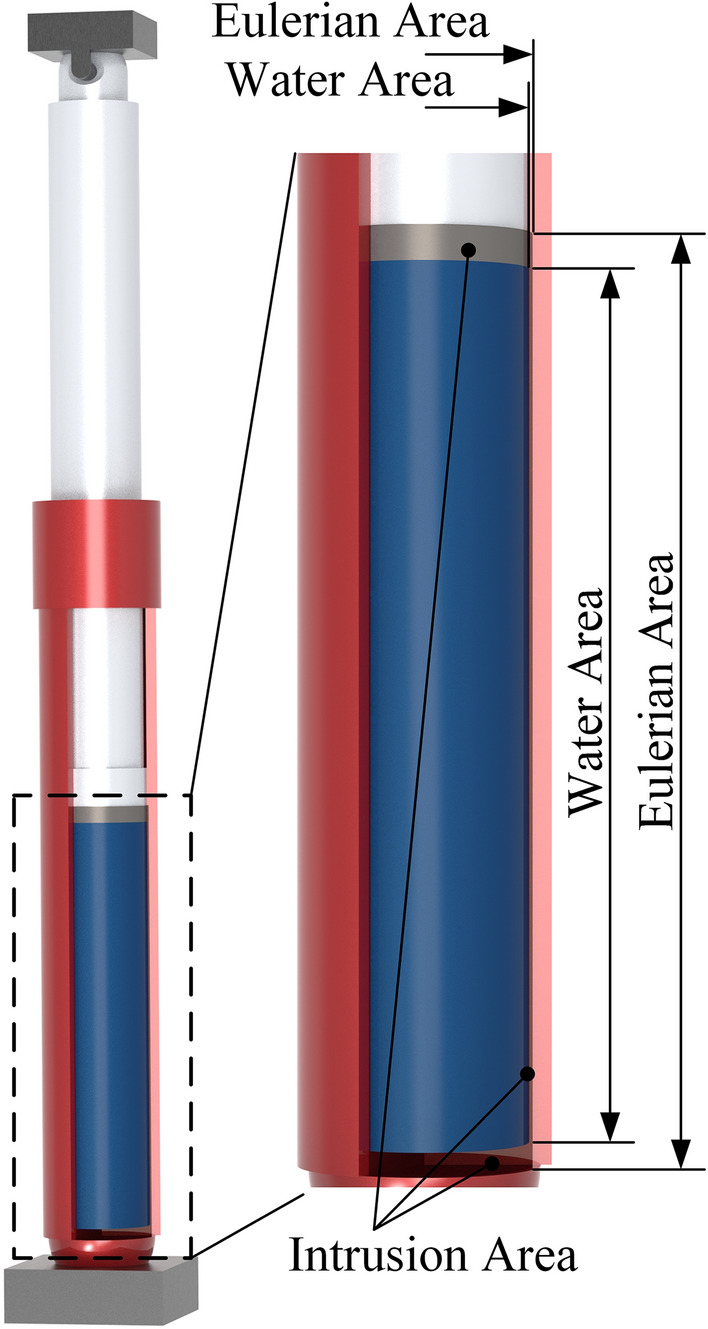
Material domain definition.

The cylinder is filled with emulsion. In the CEL algorithm, the material definition of the Eulerian domain includes the Eulerian full domain and the material domain. The Eulerian full domain represents the potential positions of the material (emulsion), and the material domain represents the actual position of the material at the initial moment. Considering that the cylinder will expand elastically during the impact process, its expansion range should be taken into account when defining the Eulerian domain. The Eulerian domain intersects with the Lagrangian domain, as shown in Fig. 5, the Eulerian domain invades the side and bottom surfaces of the inner wall of the cylinder and the end of the piston rod. This intrusion does not indicate the actual location of the material within the Eulerian domain that fills the modified area, only when the boundary of the Lagrangian domain is altered.

The material parameters of the emulsion are defined by the equation of state, which represents the functional relationship between the parameters of the material in different states. It establishes the relationship between the per unit mass internal energy *E*_*m*_, fluid pressure *p*, and current fluid density *ρ*. This research utilizes the *Mie-Gruneisen* equation of state:

14 $$f(\rho )={\rho }_{w}^{0}{c}_{0}^{2}\gamma (1-{\Gamma }_{0}\gamma /2)/{[(1-s\gamma )]}^{2}$$

15 $$g(\rho )={\Gamma }_{0}{\rho }_{w}^{0}$$

Here, ${\rho }_{w}^{0}$ represents the initial density of the water flow, ${c}_{0}$ denotes the propagation rate of the acoustic wave in the fluid, and ${\Gamma }_{0}$ stands for the material constant. The parameters are shown in Table 2.

**Table 2:** Emulsion parameters.

Density $\rho_{w}^{0}$ (kg/m^3^)	Viscosity $\eta_{w}$(N·s·m^−2^)	Mie-Gruneisen equation of state		
		$c_{0}$	*s*	$\Gamma_{0}$
980	0.001	148,000	0	0

#### Boundary conditions

**Figure 6:**
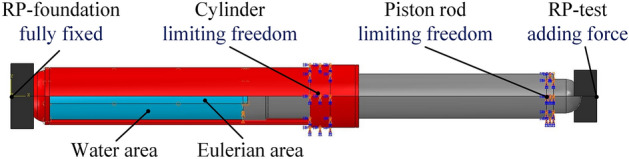
Model boundary definition.

The impact process involves numerous collisions, compressions, contacts, and other material nonlinear problems. In order to prevent the simulation process from failing to converge, the model boundary conditions are reasonably simplified. The base and indenter are defined as rigid bodies, and their motion is constrained by reference points. The base sets complete constraints, and the indenter only moves in the axial direction along the cylinder barrel. The piston rod is restricted to move only in the axial direction. As shown in Fig. 6.

**Figure 7:**
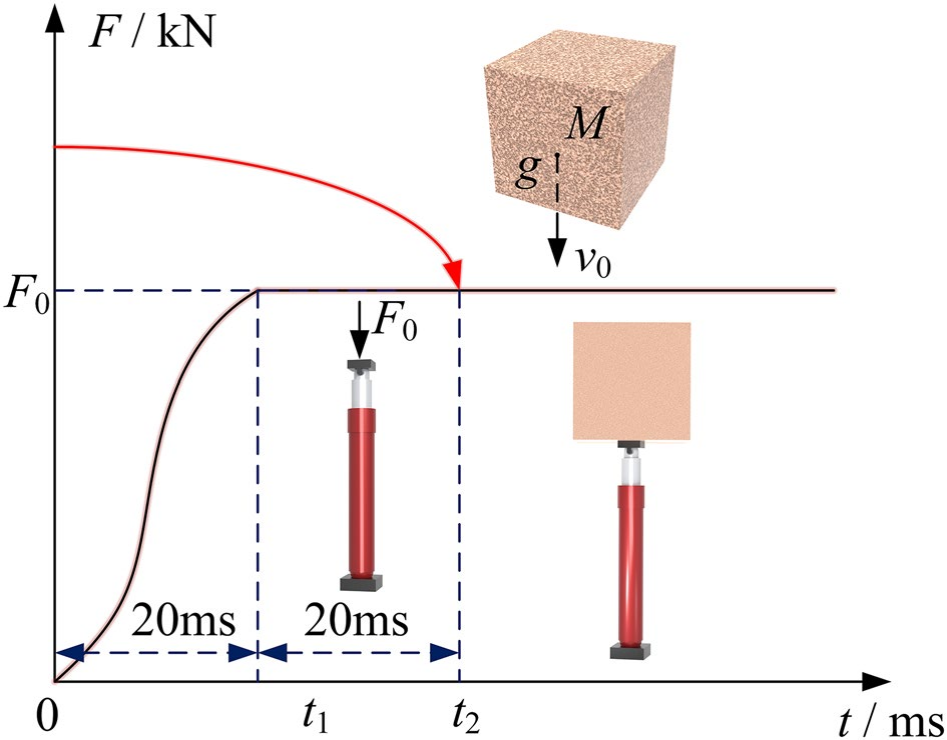
External load application curve.

In this section, the CEL fluid–solid coupling algorithm is used to simulate the impact process of a mass falling and its impact on energy conversion. According to the "Hydraulic Support for Coal Mines Part 2: Column and Jack Technical Conditions," the test conditions for center overload performance requirements^21^ state that a falling hammer with a minimum weight of 10,000 kg should be used to impact the column, in order to achieve a pressure chamber pressure that is at least 1.5 times the rated working pressure. A 20,000 kg mass was selected for impact, the impact force was measured at 1500 kN. Using Eq. (5), the column equivalent stiffness was calculated to be 53,765.70 kN/m. Equation (7) was used to calculate the dynamic load coefficient, which resulted in a value of 7.65. The kinetic energy of the mass at the time of impact was determined to be 15,456 J^22^. Using Eq. (12). Finally, the impact duration was calculated to be 33.2 ms. Load the application as shown in Fig. 7. 0–20 ms using a smooth analysis step on the column to apply the rated resistance, 20–40 ms to maintain the stability, release of elastic potential energy to weaken inertial action. After 40 ms, after the consistently maintains kept the load without releasing it. This simulates simulating column's ability column to the loads. Additionally, and at time, mark, as the mass of the impacts the impact initial allowing for the calculation of the column's the static movement. By calculating the distance of statically static column, it is determined that column, the height of the mass is 0.0137 mm its the initial velocity is 0.85 m/s.

#### Contact pair definition

The generalized contact algorithm in Abaqus/Explicit is used to track the contact between the Lagrangian unit and the Eulerian material in order to simulate the interaction between the fluid and the solid. Additionally, the algorithm allows for the setup of viscous fluid. The remaining contacts utilize the generic contact model with the penalized computational friction model, where the friction coefficient is set to 0.15.

#### Meshing

The mesh uses C3D8R hexahedral grid cells. The Euler domain is the main area of calculation, with a grid size set to 7 mm. The grid size for the remaining structural components is set to 12 mm, ensuring that the grid cells consist of at least 4 layers and enhancing the accuracy of the simulation results.

### Analysis of simulation results

**Figure 8:**
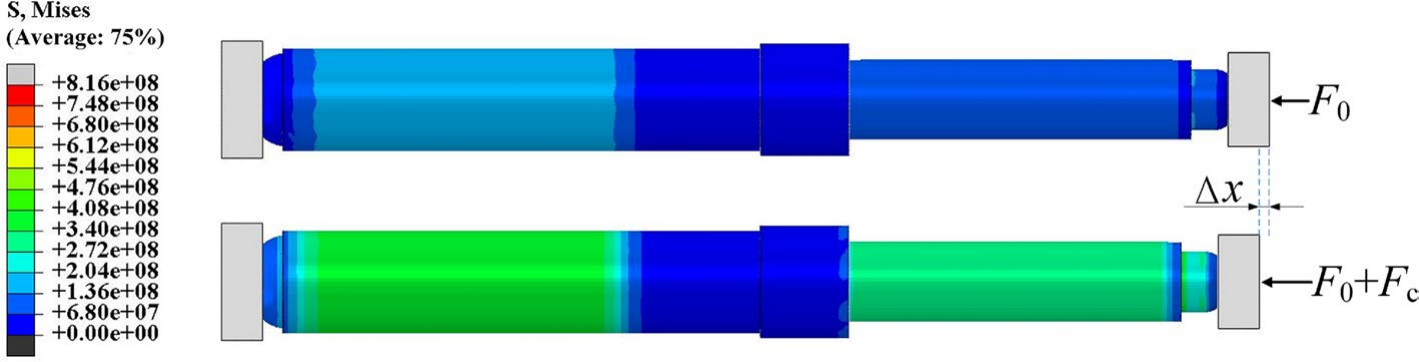
Stress cloud map of the conventional column at different times.

Figure 8 shows the stress cloud map of the column at different times. From the figure, it can be seen that the active column primarily experiences compressive stress after being impacted, while the cylinder experiences both tensile and compressive stresses. The stress value in the cylinder region is significantly higher than that in the active column region. In actual applications, the main damage to the column under the condition of no bias load is cylinder expansion, cracking, and cylinder explosion. Therefore, the cylinder is the focus of analysis.

1. Reaction force and displacement
  Figure 9 shows the curves of the reaction force of the base support, the displacement of the piston rod and the displacement of the mass over time. The column is able to maintain a relatively stable state for 39.67 ms after the loading and holding process. At this stage, the column reaches a stable state with a working resistance of one time, and the piston rod and mass have a total downward displacement of 22.10 mm and 41.64 mm, respectively. The impact process ③ can be divided into three stages: Stage I is characterized by an increase in reaction force. During this stage, the column is subjected to the impact kinetic energy of the mass, resulting in a continuous increase in the reaction force. The maximum reaction force of 2578.16 kN is reached at 66.00 ms. This stage lasts for 26.33 ms. The mass and the piston rod move together, with a total downward displacement of 23.65 mm. Stage II is characterized by a significant deceleration of the system, with the acceleration decreasing continuously. At 69.33 ms, the mass and the column reach the lowest point of displacement simultaneously, and the value of the reaction force decreases in a parabolic pattern, reaching 2516.76 kN at this stage. Stage III is the phase following the lowest point, during which the mass and the column generate a 13.78 mm rebound and experience significant separation at 101.00 ms. The reaction force in this process decreases linearly until it stabilises at around 1000 kN. Process ④ is when the height of the piston rod and the reaction force of the column reach a stable state. The mass continuously moves upward due to the rebound force, completing one impact of the mass on the column. The whole impact process takes 61.33 ms. The piston rod has a displacement difference of 10.24 mm before and after the impact. The reason for this is that there is a significant elastic expansion in the cylinder after the impact. This expansion phenomenon occurs at the end of the piston rod, where there is higher stress, causing some liquid to leak from the side of the piston rod end. As a result, there is a significant displacement difference.

**Figure 9:**
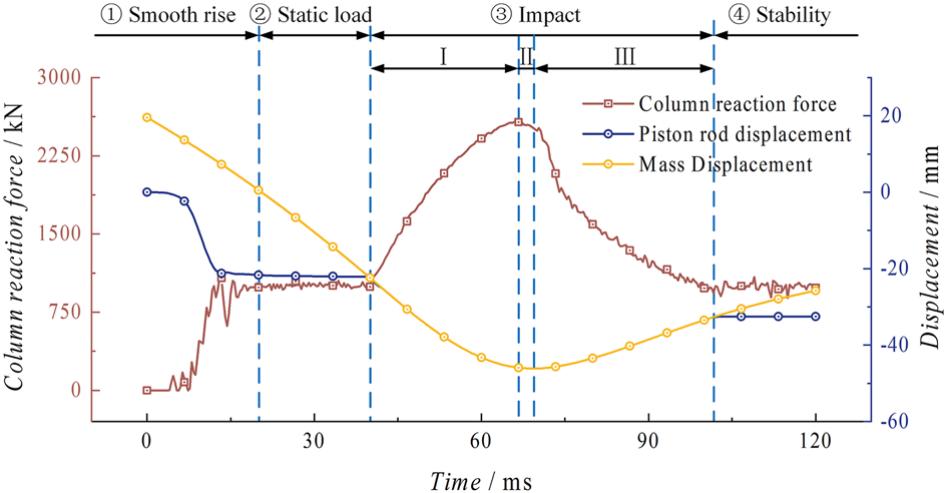
Impact process reaction force and displacement curve of the conventional column.
2. Cylinder stress–strain and expansion
  As shown in Fig. 10, the cloud map displays the scaled 100 times stress and strain characteristics of the peak point of the cylinder. There are significant extreme points of stress at the bottom of the cylinder and the end of the piston rod, with extreme values reaching 368.32 MPa and 379.48 MPa, respectively. The stress in the middle section of the cylinder is stable. The variation in strain in the cylinder is consistent with the trend as the variation in stress. There are significant extreme values of strain at the bottom of the cylinder and the end of the piston rod, with strain values of 1702.66 με and 1774.46 με, respectively. The strain in the middle section of the cylinder is also stable. Figure 11 shows the radial expansion map of the outer surface of the cylinder at different heights at the moment of the peak point of impact. The two extreme points are 0.177 mm and 0.186 mm, and the maximum expansion accounts for 0.042% of the total length. The radial expansion gradually increases in the middle section of the cylinder. Since the bottom region of the cylinder is a closed end, the solid at the bottom provides an effective bending moment for the radial expansion of the cylinder, resulting in a significant indentation. On the other hand, the upper end of the cylinder is an open structure, and its tightening effect is not significant.

**Figure 10:**
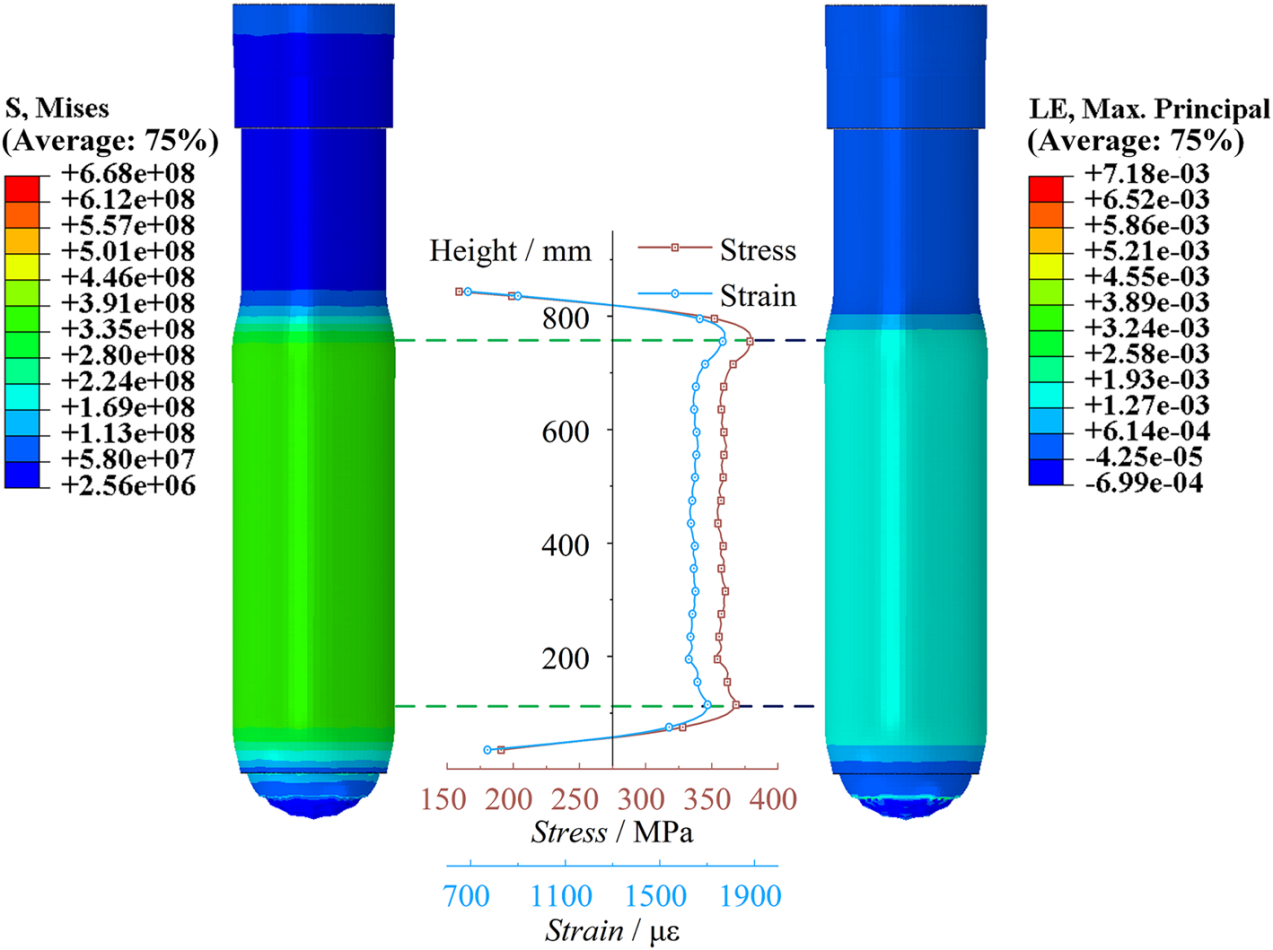
Cylinder stress–strain diagram of the conventional column.

**Figure 11:**
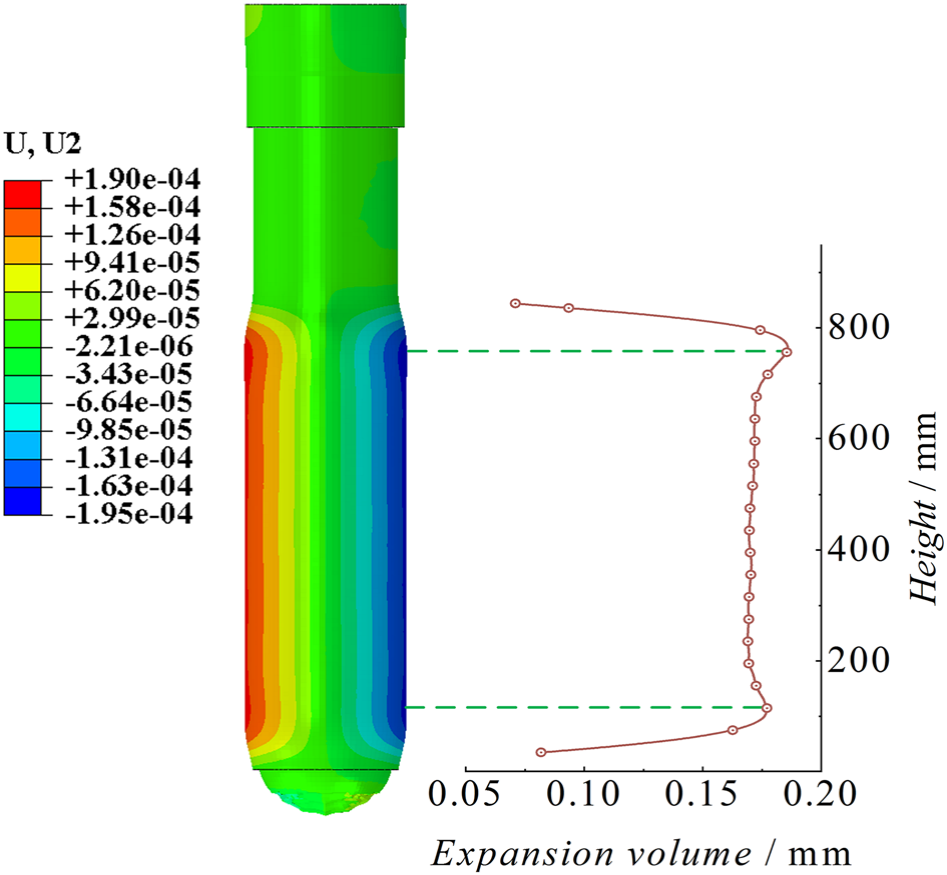
Cylinder radial expansion of the conventional column.
3. Velocity of motion
  Figure 12 shows the velocity curve of the piston rod and the mass. At time *t*_*1*_, which is 39.67 ms before, the mass accelerates due to the force of gravity, while the piston rod remains stable after oscillating through processes ① and ②. During stage ①, the piston rod velocity tends to increase and then decrease under the influence of the external force. It gradually stabilizes at 0 m/s in stage ②. The first peak velocity point is generated by the smooth rise of the external force and will return to 0 m/s before impact. The generation of this point will not affect the simulation. After the collision, the piston rod reaches time* t*_*2*_ after 2.33 ms, with a peak velocity of 1.91 m/s. It then oscillates at a high speed and maintains a consistent velocity with the mass, starting to decelerate until reaching time *t*_*3*_ at 69.33 m/s, where both velocities become 0 m/s. At time* t*_*4*_, specifically at 101.00 ms, there is a noticeable disparity in the velocities of the two objects. The mass continues to decelerate, while the velocity of the piston rod quickly drops to nearly 0 m/s and remains stable.

**Figure 12:**
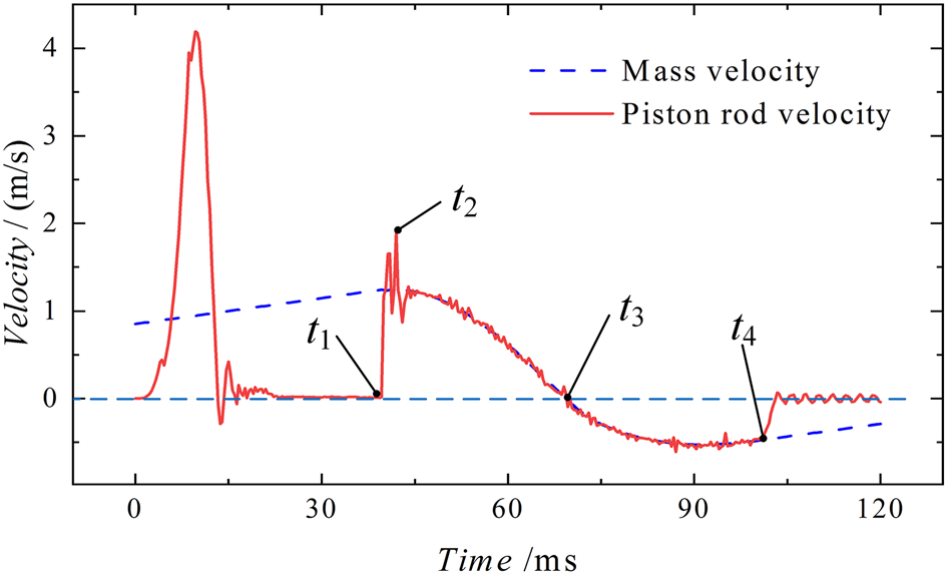
Piston rod and mass velocity diagram of the conventional column.
4. Energy absorption
  As shown in Fig. 13, the graph depicts the reaction force displacement and energy absorption curve of the column after impact. After the displacement reaches − 22.39 mm, the force–displacement curve shows a linear upward trend in the initial stage, with a growth rate of 67.56 kN/mm. The reaction force remains almost stable at the − 58.25 mm position, while the energy absorption continues to increase linearly. The maximum energy absorption occurs at − 46.12 mm, reaching 43.63 kJ. The total energy in this process includes the continuous action of the rated pressure, the gravitational potential energy after the impact of the heavy object, and the total kinetic energy before the impact of the heavy object. Therefore, the energy absorption exceeds the impact energy set for the heavy object.

**Figure 13:**
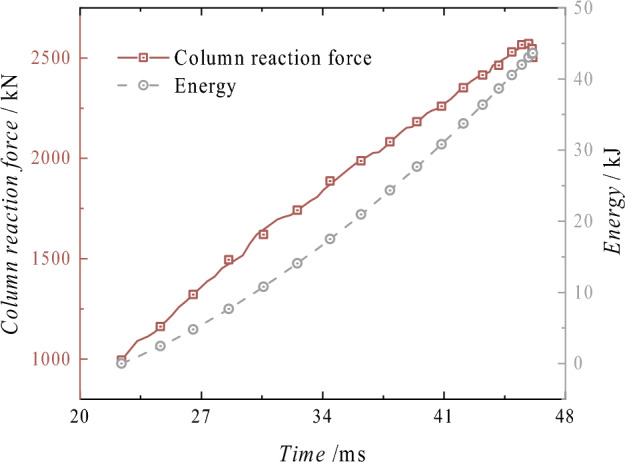
Reaction force–displacement and energy absorption curves of the conventional column.

## Conventional column impact test verification

### Test machine system and specimens

**Figure 14:**
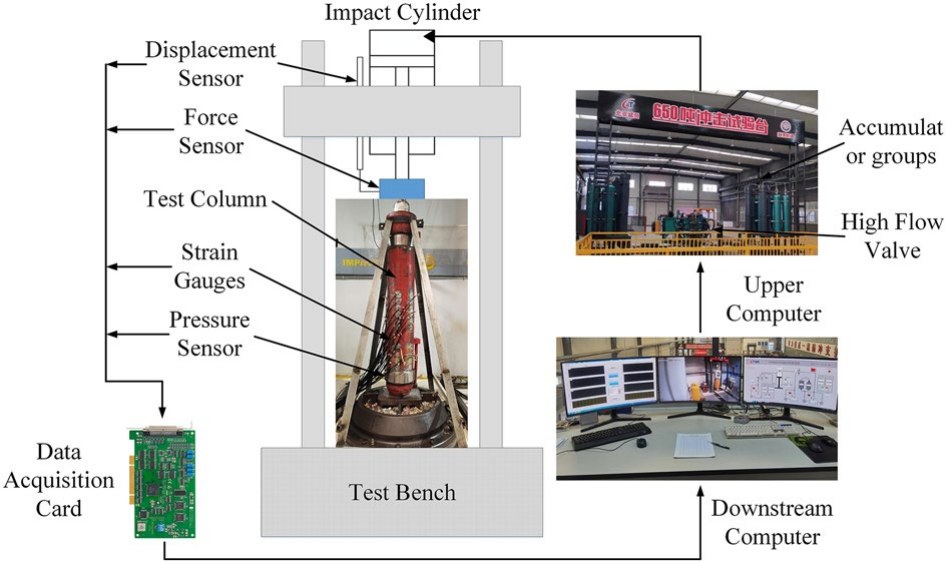
Test machine systems and specimens.

The rock burst exhibits characteristics such as high impact energy, short conversion time between static and dynamic loads, and high impact velocity. At present, the primary methods for researching anti-impact support devices rely on theoretical calculations and numerical simulations^23–25^. However, there is a lack of research on static-dynamic combination loading tests for anti-impact support devices. Our team has developed the first 6500 kN hydraulic impact test machine in China to meet different impact requirements^26,27^. The test machine system and specimen are shown in Fig. 14.

1. Test specimen: It adopts the 2.2 section single-piston rod double-acting hydraulic column with a cylinder diameter of 180 mm. Both ends of the column are spherical.
2. Test machine system: It utilizes a 6500 kN static-dynamic combination hydraulic impact test machine, capable of performing stepless pressure regulation for static loads and conducting high-energy impact tests for dynamic loads to simulate underground working conditions. The advantage of this system is that the static-dynamic load conversion time does not exceed 50 ms, which can meet the time requirements for simulating rock burst occurrences.

### Monitoring parameters

The test machine can monitor parameters such as column reaction force, liquid pressure, impact displacement, and cylinder strain. Each parameter is collected using a 20,000 Hz high-speed acquisition device. In order to measure the extent of strain on the cylinder, four rows of strain gauges are attached to the outer surface of the cylinder. The second and fourth rows are positioned at a 60° angle relative to the first row, allowing for comprehensive measurement of cylinder strain.

### Test process

The lower end of the impact cylinder of the test machine is equipped with a spherical socket surface, and the upper end of the piston rod head is connected to the lower end spherical socket surface of the test machine impact cylinder. Before loading the test specimen, the column is extended to a position of 800 mm. During static-dynamic combination loading, the static pressure simulates the ground stress on the column, and the dynamic load simulates the energy release during a rock burst occurrence. The test considers the dynamic response characteristics of the column in the absence of a safety valve. The test is conducted with a static load of 1 times the rated working resistance and a dynamic load of 2.5 times the rated working resistance for verification.

### Results analysis and comparative verification

**Figure 15:**
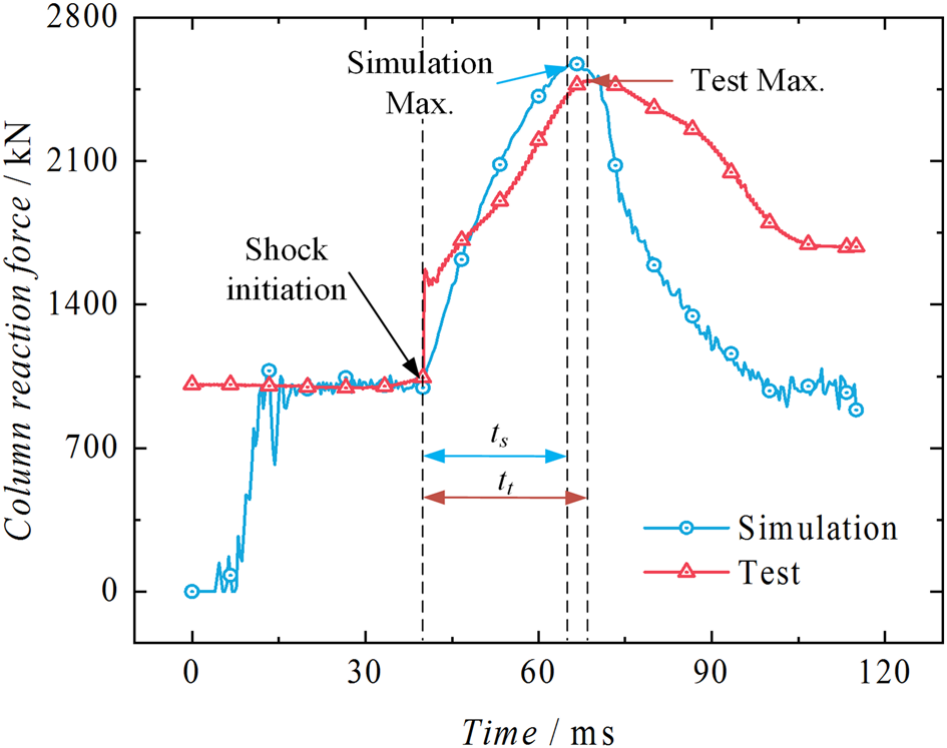
Test-simulation reaction forces comparison.

As shown in Fig. 15, the test-simulation reaction force curves are being compared. The two impact curves are defined at 40 ms as the starting point of impact. The initial pressure of the experiment stabilizes around 1009 kN with slight fluctuations and reaches its first peak of 2494.79 kN at 68.40 ms. The pressure boosting stage, tt, lasts for 28.40 ms. The simulated peak value is 2578.16 kN, and the pressure boosting stage, ts, lasts for 26.33 ms. The error of the peak impact point is 3.34%, and the error of the impact time is 7.29%, which falls within an acceptable range.

**Figure 16:**
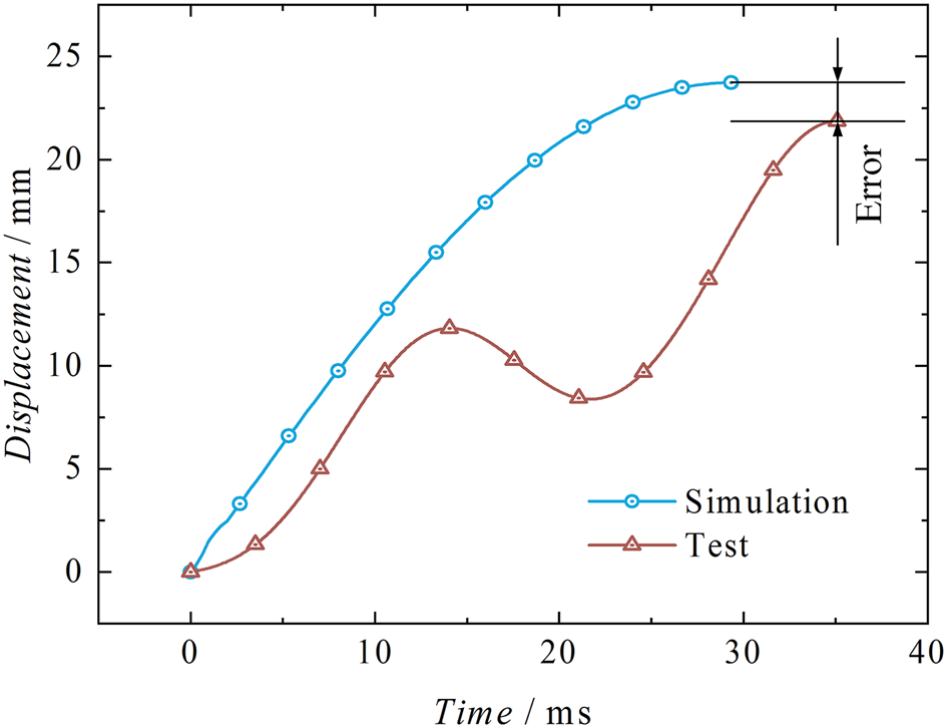
Test-simulation displacement comparison.

**Figure 17:**
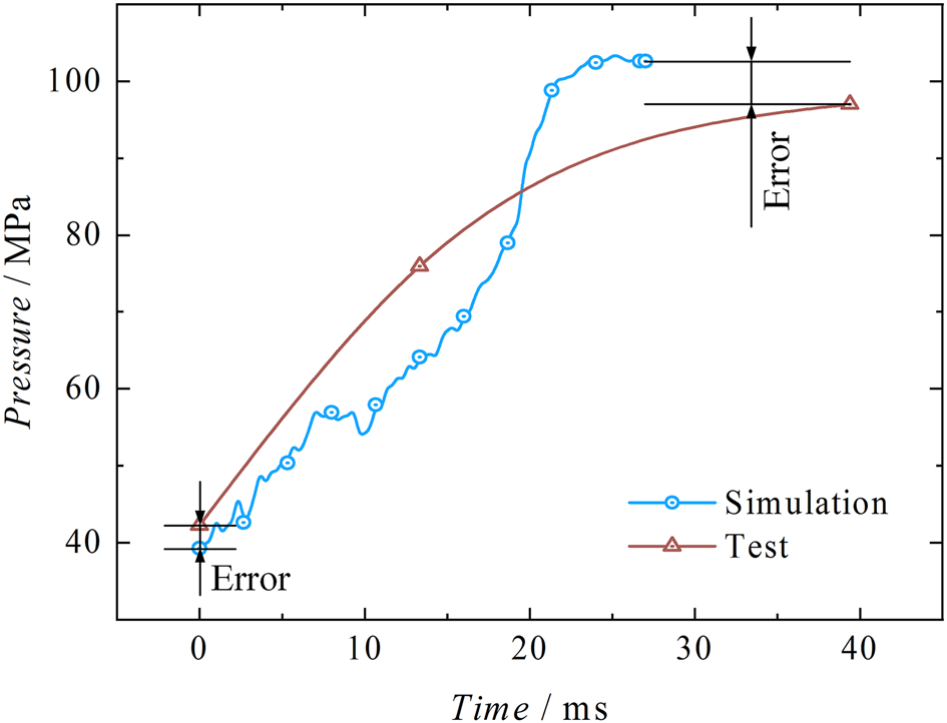
Test-simulation pressure comparison.

From the test-simulation displacement comparison curves in Fig. 16, it is evident that both the test and simulation exhibit a significant upward trend in displacement. The simulation compresses the liquid by a total of 23.73 mm, effectively demonstrating the phenomenon of liquid compression. However, compared to the test, the compressibility in the simulation is more significant. The compression amount in the test is 21.87 mm, with an error of 8.50%. Figure 17 shows the test-simulation liquid pressure comparison curves. During the pressure boosting process, both the simulation and test show an increasing trend in pressure. The simulation process is more affected by stress waves, which leads to more significant pressure fluctuations. The highest simulated liquid pressure is 103.35 MPa, occurring at 27.00 ms during the pressure boosting stage. The highest test liquid pressure is 96.99 MPa, occurring at 39.38 ms during the pressure boosting stage. Both of them exhibit a delay effect caused by stress waves, with the delay effect being more pronounced in the test, with an error of 6.55%.

**Figure 18:**
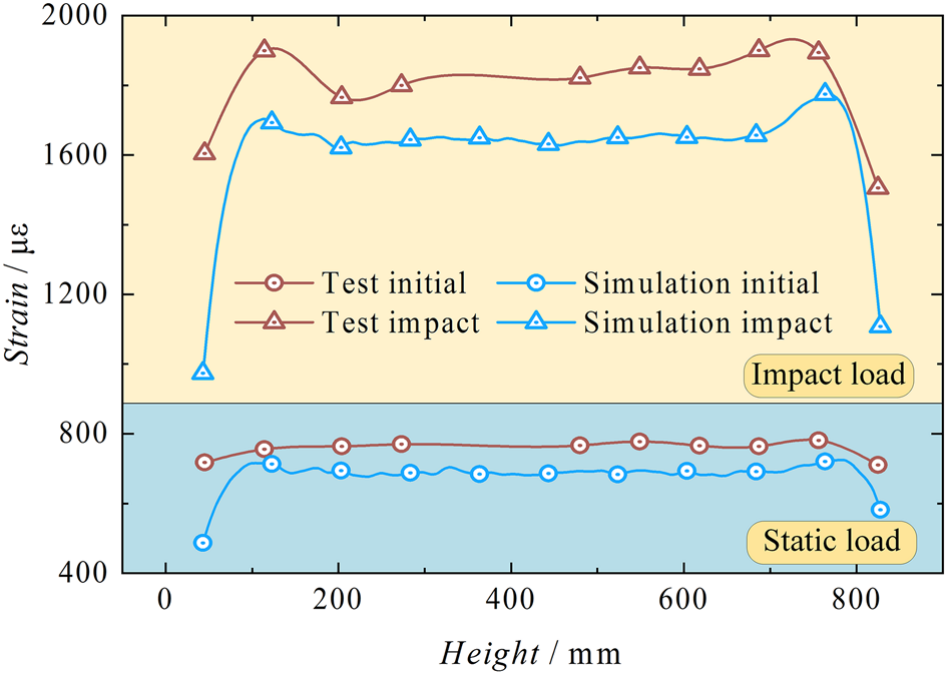
Test-simulation strain comparison.

By comparing the strain values on the outer wall of the cylinder at different times, a baseline is established at the axial height of 50–850 mm on the outer wall of the cylinder. The strains at different baselines are plotted and compared with the test strains, as shown in Fig. 18. The simulation results show a similar trend to the test results under two different conditions, with significant peak points in strain observed at the bottom of the cylinder and the end of the piston rod. The simulated values are slightly lower than the test values. Table 3 shows that the strain error rate between test and simulation is within reasonable limits.

**Table 3:** Test-simulation strain error comparison.

	Cylinder bottom strain peak /με		Cylinder top strain peak /με	
	Initial	Impact	Initial	Impact
Simulation	715.24	1702.66	725.11	1774.46
Test	755.77	1899.52	780.79	1900.10
Difference	40.53	196.86	55.68	125.64
Error rate (%)	5.36	10.36	7.13	6.61

**Figure 19:**
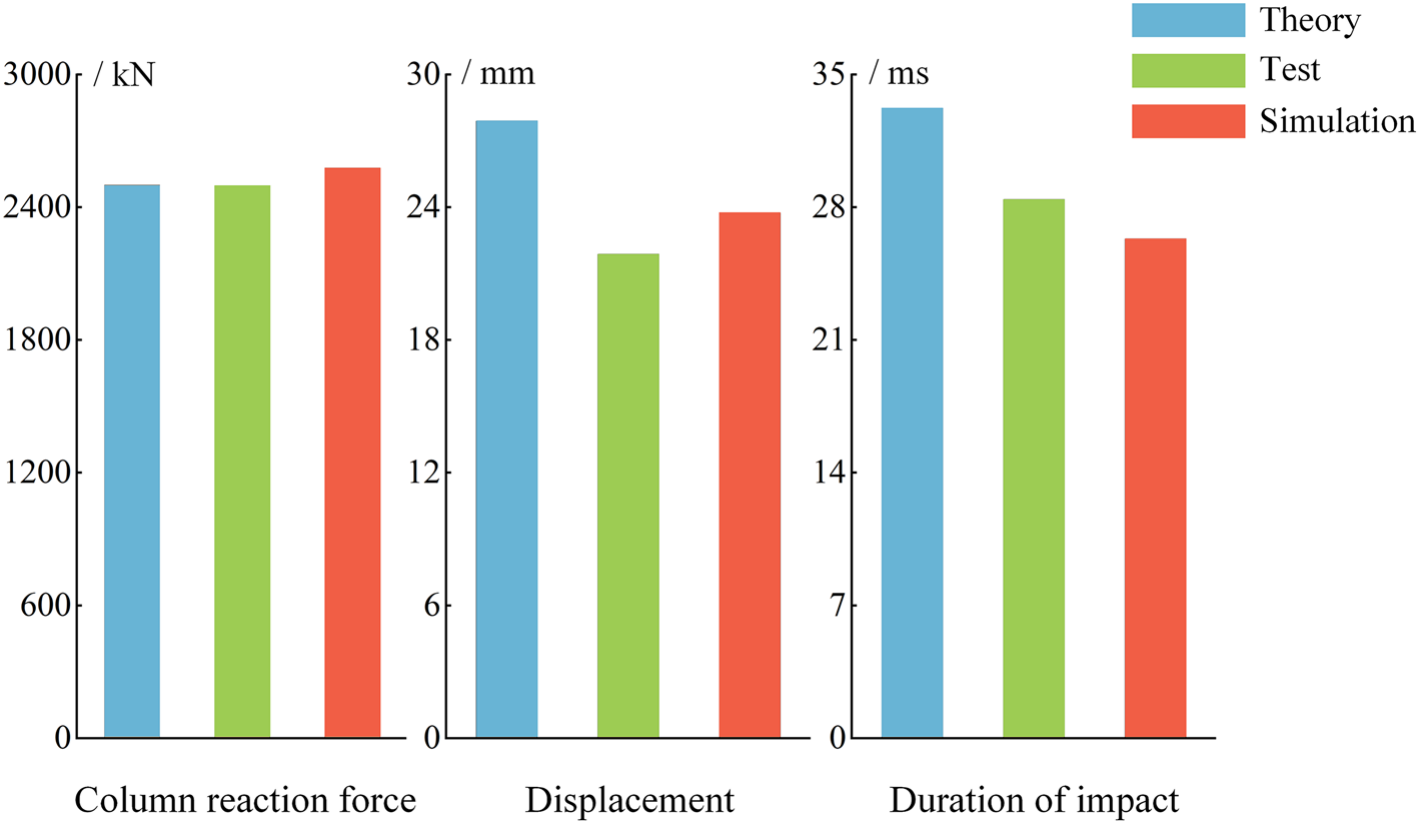
Theory-test-simulation comparison diagram.

Based on a comparative analysis of the theoretical, experimental, and simulated results of the support reaction force, impact displacement, and impact time, as described by Eqs. (6), (8), and (11) in "Conventional column impact analysis" section, as shown in Fig. 19, it is evident that the variations among the three results for different parameters are minimal. The maximum error in the comparison is not exceed 25%. The main reason for the error is the complexity of the testing environment. The theoretical analysis and numerical simulation have simplified certain factors. The error is within an acceptable range. The CEL algorithm has a positive impact on reproducing test results, and this algorithm has a certain level of accuracy and credibility in simulating column impact.

## Analysis of the bearing characteristics of energy-absorbing column under rock burst

**Figure 20:**
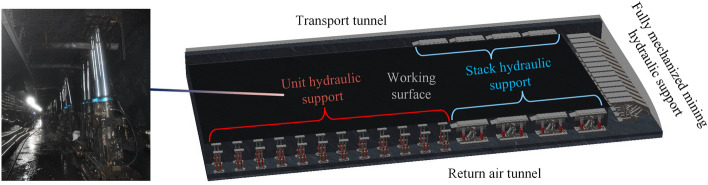
3102 working face support layout plan.

Some coal mines in China have been affected by long-term rock bursts, and currently, there are 148 mines in China experiencing rock bursts. Taking the 3102 working face of Menkeqing Coal Mine as an example, the support layout is shown in Fig. 20. The coal body impact propensity test result of the relevant unit is strong impact propensity. Long-term monitoring data shows that the working face has experienced multiple high-energy events, primarily characterized by roof subsidence, rib deformation, floor heave, and support damage. Among them, the most severe dynamic event had an energy level of 107, causing the closure of the return air tunnel within a range of 40 m ahead, coal outburst in the 40–90 m area, roof convergence of about 2 m, and production stoppage. In order to prevent tunnel collapse and damage to support equipment, some mines have now adopted energy-absorbing anti-impact supports, which have yielded positive outcomes.

### Dynamic characteristics of energy-absorbing devices

**Figure 21:**
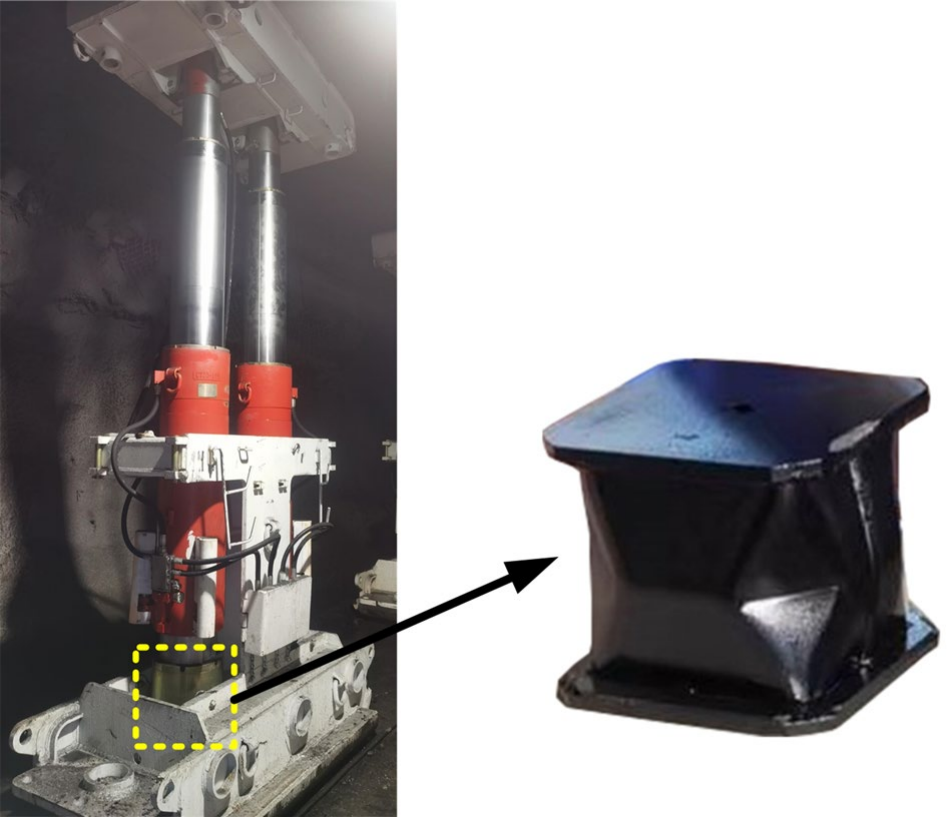
Energy-absorbing anti-impact hydraulic support.

The design principle of energy-absorbing supports is to preserve the overall structure by sacrificing a small local component. They should possess the characteristics of "high resistance" and "quick yielding". That is, under quasi-static loads, the energy-absorbing device can support the load force borne by the column, while undergoing minimal elastic deformation itself. When dynamic loads occur, the speed of the device exceeds the opening velocity of the safety valve, causing the column to exhibit variable rigidity. The initiation point of the energy-absorbing device's deformation is controlled by force. In other words, when the load force reaches the yield point of the energy-absorbing device, it immediately undergoes yielding and compression, regardless of the velocity. At the same time, the yielding process can provide enough time for the safety valve to open. At the current stage, some domestic mines have adopted energy-absorbing anti-impact supports, as shown in Fig. 21, with good anti-impact effects.

**Figure 22:**
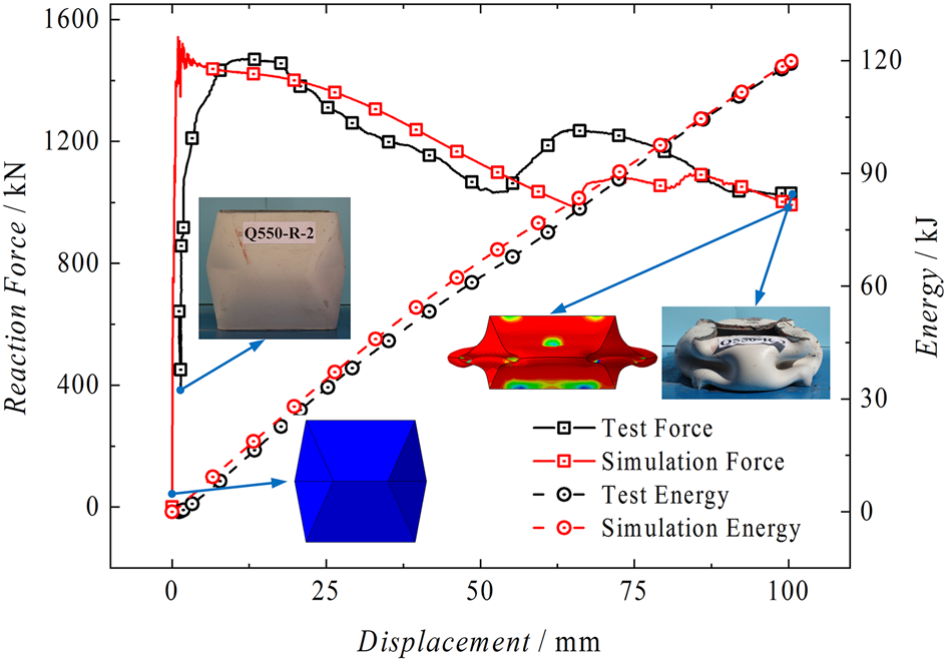
Energy absorption device test-simulation comparison.

Pre-folded energy-absorbing devices have been put into use in some mines. The design concept is based on the pre-compressed folding of square tubes to create a plastic hinge. This allows the structural devices to experience regular plastic deformation and absorb the energy generated by the induced plastic deformation. The energy-absorbing device is made by welding two plates together after pre-compressing them in accordance with the pre-designed folds. The overall appearance consists of two symmetrical sections, each containing four completely identical trapezoids and isosceles triangles. The energy-absorbing device has a side length of 180 mm, a plate inclination angle of 78°, a total height of 176 mm, and is made of Q550 material. It is hot-pressed, preheated, welded, stress-relieved, and processed into test pieces. A rigid testing machine is used to compress the test pieces, and a comparison is made with the ABAQUS Explicit module for explicit dynamic simulation calculation. The force–displacement curves and energy curves of both are shown in Fig. 22. From the figure, it can be seen that the simulation has a high accuracy compared to the test. Both the test and the simulation show significant yield points of 1470.83 kN and 1543.14 kN, respectively, with an error of 4.92%. After the yield point, there is a significant resistance reduction process, with the lowest values being 1031.55 kN and 979.49 kN, respectively, with an error of 5.05%. Within the effective compression stroke range of 100 mm, both the test and the simulation show a linear increase in energy absorption, with total energy absorption of 119.25 kJ and 119.87 kJ, respectively, with an error of 0.52%. Therefore, it can be seen that in the research of pre-folded energy-absorbing devices, the use of explicit dynamic simulation to reproduce the mechanical process of the energy-absorbing device has good reproducibility and high accuracy, and it has strong reference value in the simulation study of energy-absorbing columns.

### Analysis of load-bearing characteristics of energy-absorbing columns

**Figure 23:**
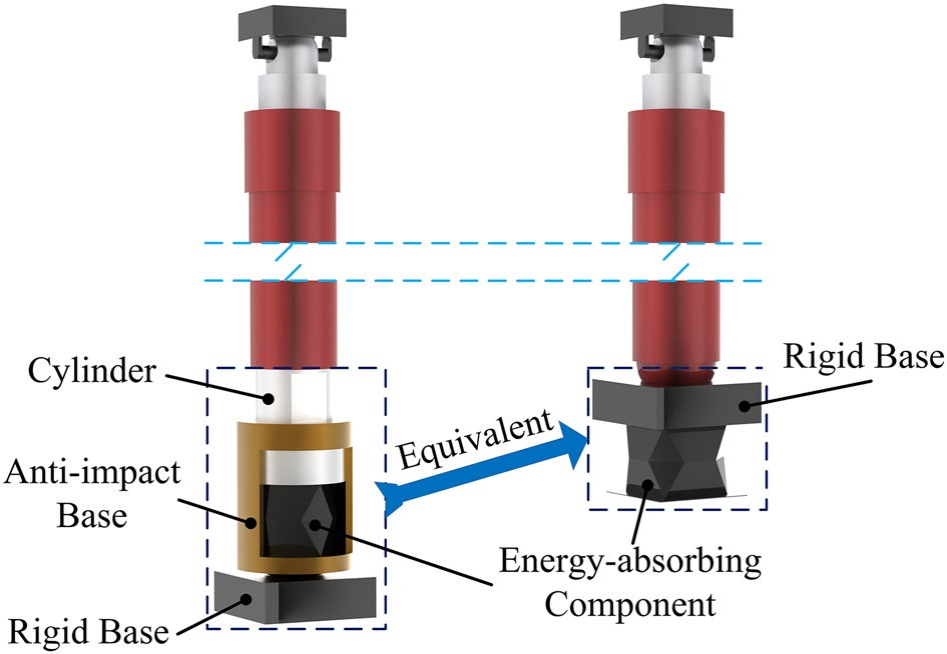
Equivalent simplification.

**Figure 24:**
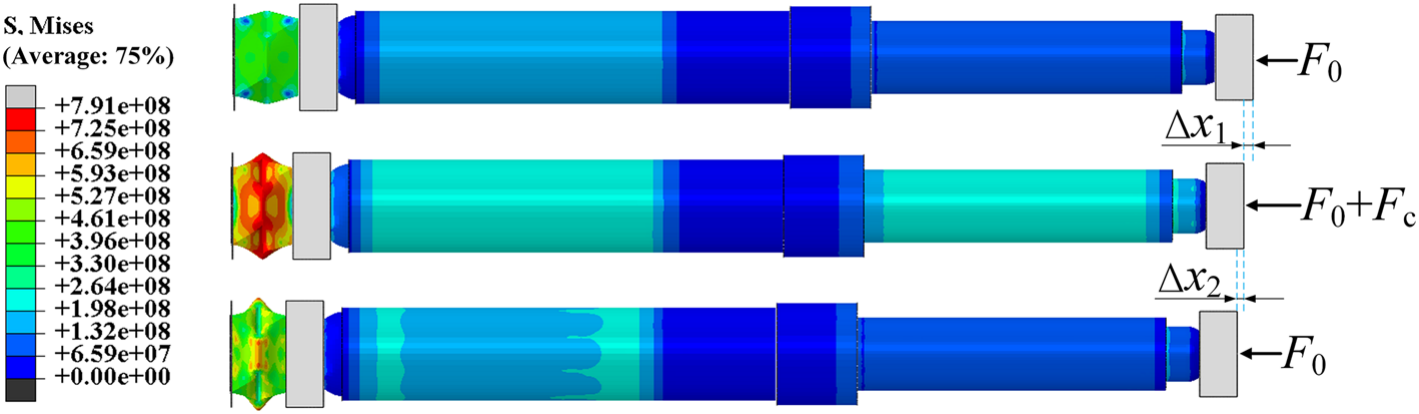
Stress cloud map of the energy absorption column at different times.

The energy-absorbing column is modeled. Taking into account a simplified contact model, both the anti-impact base and the bottom end of the column are rigid. The equivalent simplification of the column is shown in Fig. 23. Figure 24 shows the stress cloud diagram of the energy-absorbing column at different stages. Under quasi-static load conditions, the stress on the piston rod and cylinder is relatively small, while the stress on the energy-absorbing device is significant but has not reached the yield point, and the system remains in a stable state. When the impact occurs, the reaction force continues to increase until the moment of the peak reaction force. The stress on the piston rod and cylinder increases significantly, causing the energy-absorbing device undergoes buckling deformation, which results in a displacement of *Δx*_*1*_ for the system. After the mass separates from the column, the stress on the piston rod and cylinder decreases, and the stress on the energy-absorbing device decreases to below the yield point of the material, ensuring stability. The total effective displacement of the system during the entire impact process is *Δx*_*1*_ + *Δx*_*2*_.

1. Reaction force and displacement
  As shown in Fig. 25, the column goes through the smooth rising stage ① and the static loading stage ②. At 40.00 ms, it enters the impact stage ③ after being impacted by the mass. Based on the dynamic process of the column, this stage can be divided into four parts: Stage I: The impact kinetic energy of the mass continuously increases the reaction force of the column. At 46.00 ms, the support reaction force of the column is 1511.37 kN, exceeding the peak load of the energy-absorption device for the first time. At this time, the energy absorption device begins to undergo buckling deformation. Stage II: Under the inertia force of the mass impact, the column reaches its peak load at 59.67 ms, which is 1739.70 kN. This peak load occurs 19.67 ms after the collision. During this process, the piston rod and the mass undergo a displacement of 20.75 mm, and the rate of increase in reaction force decreases significantly. Stage III: The reaction force of the column decreases as the energy-absorption device controls the buckling deformation. This results in a significant decrease in the velocity movement of the piston rod and the mass. Stage IV: At 100.67 ms, the piston rod and the mass move together to the lowest point. Then, the piston rod pushes the mass to move in the opposite direction. At this time, the reaction force of the column rapidly decreases until 113.33 ms, when the piston rod and the mass completely separate. The piston rod remains in a stable state, and the reaction force eventually stabilizes at around 1000 kN. In Stage ④, the system tends to a stable state, and the mass continues to move upward under the action of the rebound force, completing one impact of the mass on the energy-absorption column. The entire impact process lasts for 73.33 ms, during which the energy-absorption device undergoes partial buckling deformation.

**Figure 25:**
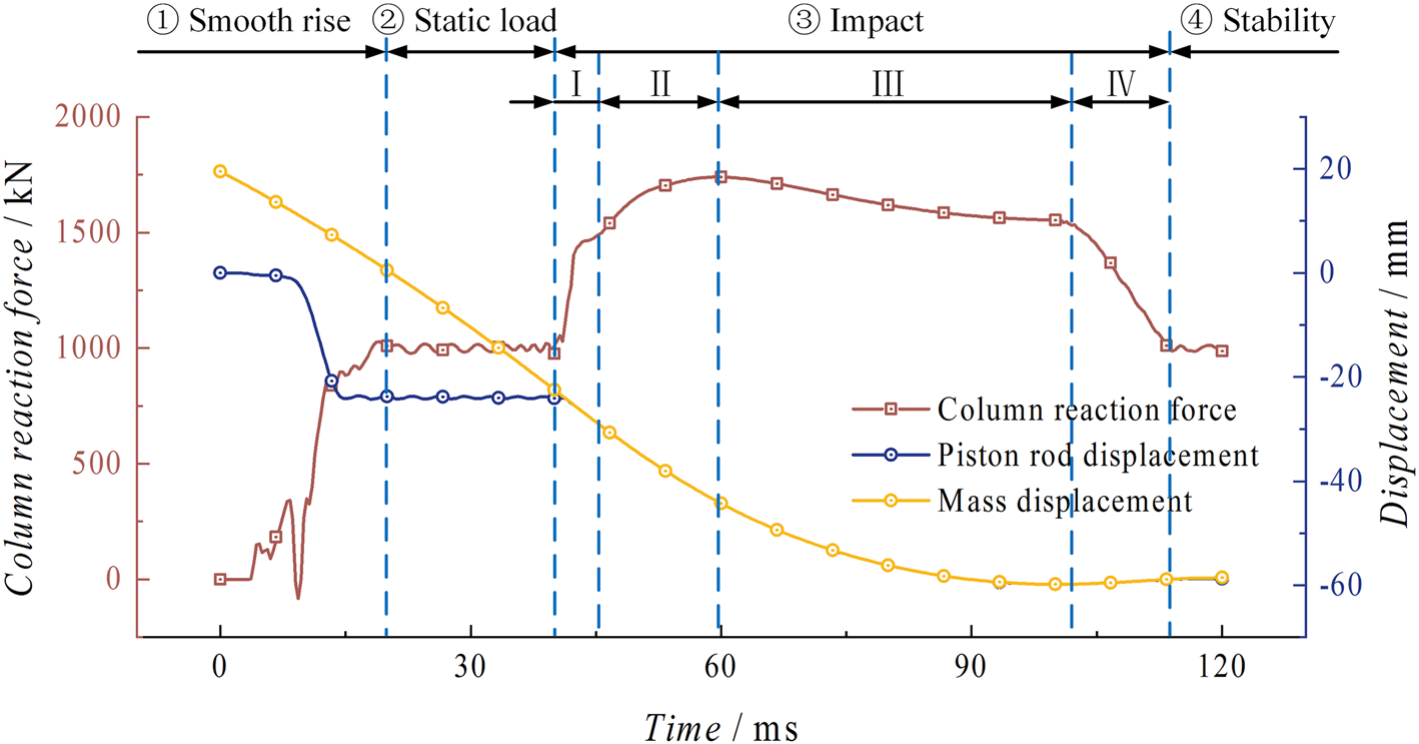
Impact process reaction force and displacement curve of the energy absorption column.
2. Cylinder stress–strain and expansion
  As shown in Fig. 26, the stress–strain diagram of the cylinder at the moment of peak impact reaction force of the energy-absorbing column is shown. The stress peaks are significant at the bottom of the cylinder and the end of the piston rod, with stress values of 242.933 MPa and 250.564 MPa, respectively. The stress value at the end of the piston rod is slightly higher than that at the bottom of the cylinder. The stress value in the middle of the cylinder changes steadily and slightly increases with height. The variation trend of strain in the cylinder is consistent with the variation trend of stress. The strain peaks at the bottom of the cylinder and the end of the piston rod are 1115.92 με and 1158.37 με, respectively. As shown in Fig. 27, the cylinder radial expansion diagram is shown. It can be seen from the diagram that the expansion amount also has obvious extreme points at the bottom of the cylinder and the end of the piston rod, but these extreme points are not clearly reflected. The radial expansion in the middle section of the cylinder increases linearly with height. The two extreme expansion amounts are 0.117 mm and 0.121 mm, respectively. The maximum expansion accounts for 0.028% of the total length.

**Figure 26:**
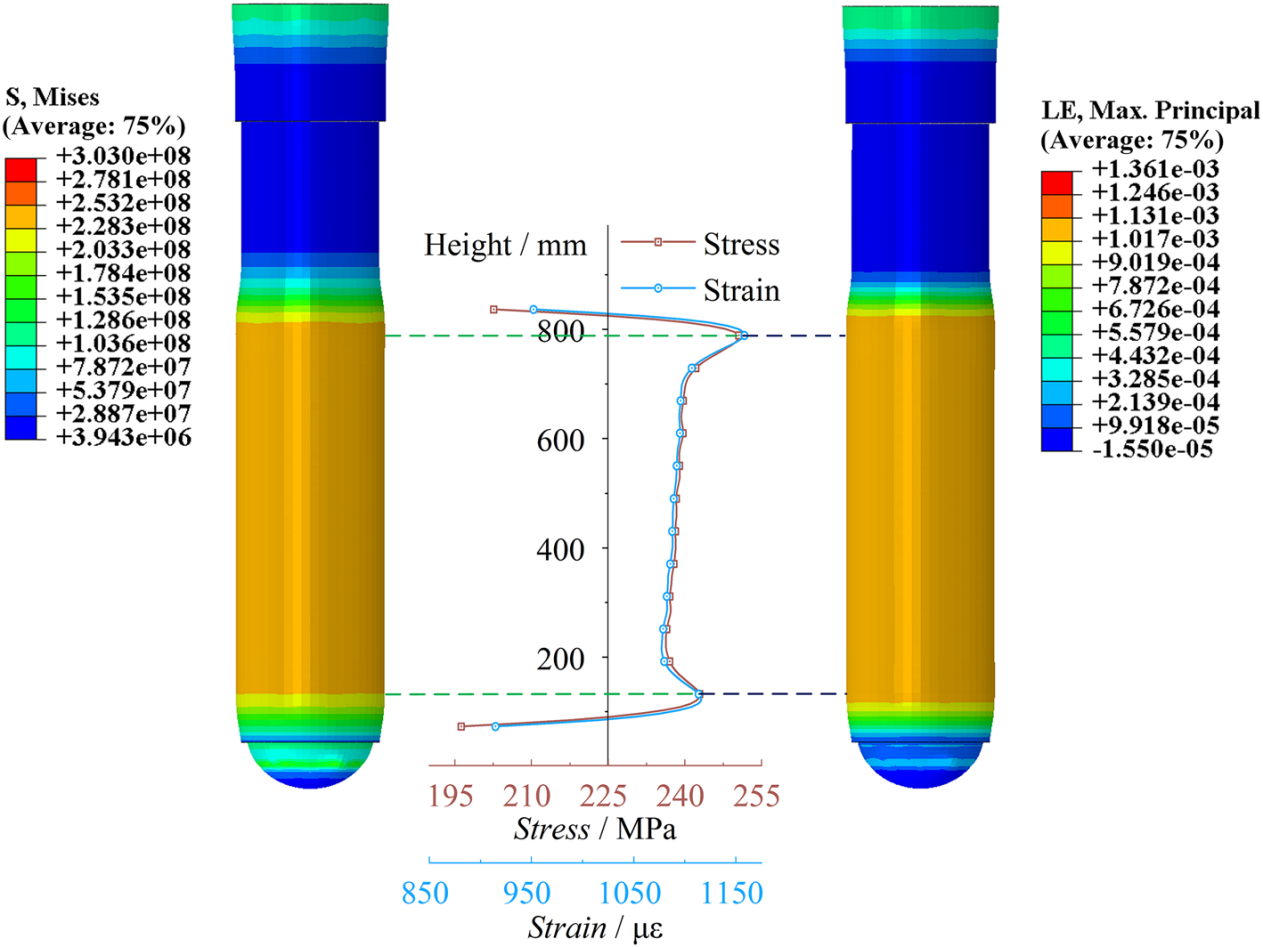
Cylinder stress–strain diagram of the energy absorption column.

**Figure 27:**
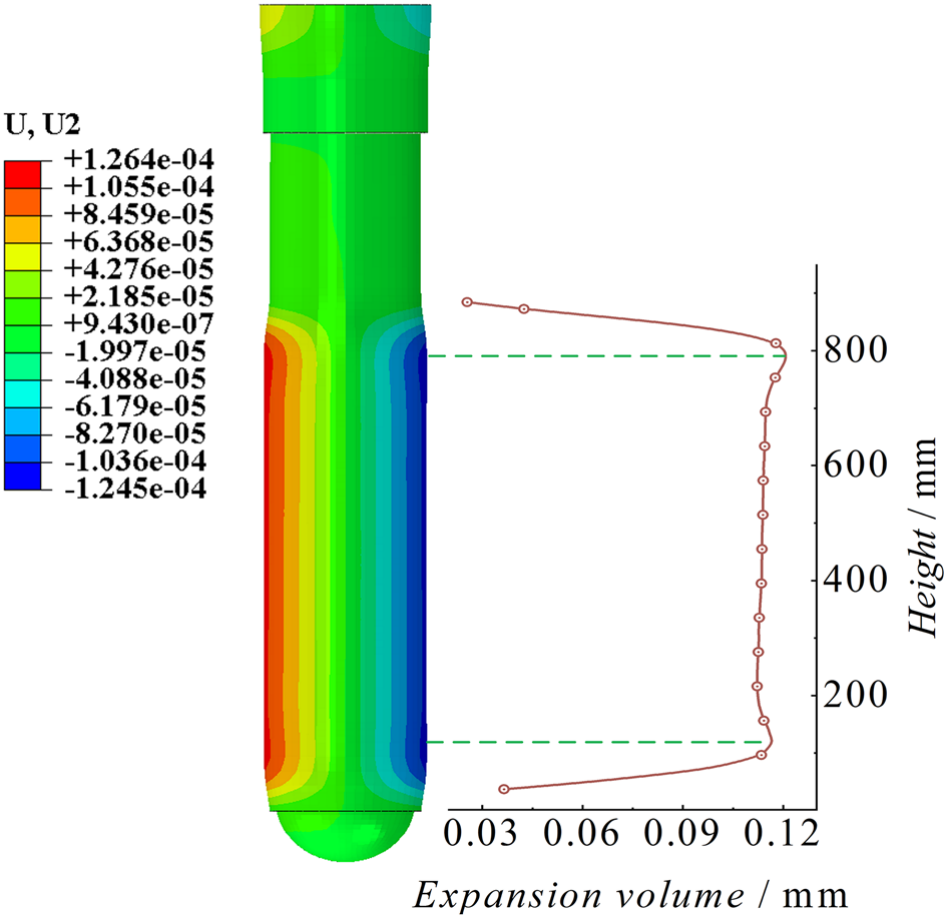
Cylinder radial expansion of the energy absorption column.
3. Velocity of motion
  The diagram in Fig. 28 shows the velocity curve of the energy-absorbing column piston rod and the mass. After the stable application of the load in stages ① and ②, an impact occurs at time *t*_*1*_. At 1.67 ms after the impact, the velocity reaches its peak point *t*_*2*_, at 1.45 m/s. After the contact oscillation, the mass moves together with the piston rod and maintains a constant velocity until they both reach a velocity of 0 m/s at time *t*_*3*_, which is 100.67 ms after the impact. The subsequent process is the same as the conventional column impact process, where the mass undergoes reverse motion and the velocity of the piston rod remains steady near 0 m/s.

**Figure 28:**
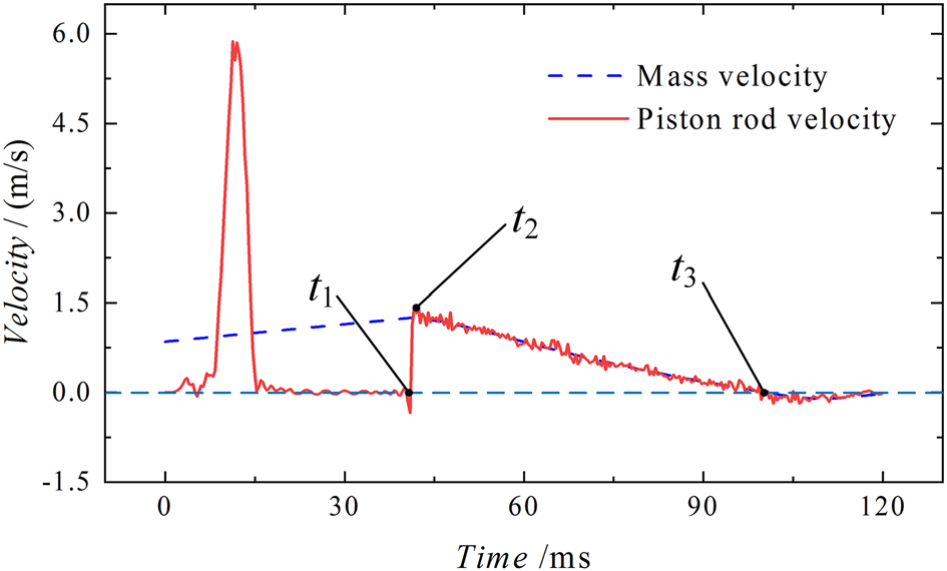
Impact process velocity curve of the energy absorption column.
4. Energy absorption
  As shown in Fig. 29, it illustrates the displacement-reaction force and energy absorption curve of the energy-absorbing column following impact. The reaction force of the column is significantly influenced by the energy-absorbing device after impact, and its variation trend is consistent with that of the energy-absorbing device. The reaction force initially increases and then decreases with displacement, and the fluctuation range of the force throughout the entire process is small. The energy absorption exhibits a linear upward trend, with the maximum energy absorption recorded at 58.23 kJ at − 59.82 mm. The total energy of this process includes the continuous action of the rated pressure, the gravitational potential energy after the mass impact, the total kinetic energy before the mass impact, and the energy absorption of the energy-absorbing device. Comparing the overall energy absorption of the energy-absorbing column with the energy absorption of the energy-absorbing device in Fig. 30, it can be observed that the energy-absorbing device contributes to energy absorption with a total of 47.94 kJ, accounting for 82.33% of the total energy absorption of the column. The effective displacement of the energy-absorbing column is 37.38 mm, with the plastic deformation of the energy-absorbing device is 29.76 mm, which accounts for 79.62% of the total displacement. The addition of the energy-absorbing device can effectively reduce the energy absorbed by the active column itself. It replaces the displacement of the column under impact conditions with the bending deformation displacement of the energy-absorbing device, thereby improving the protection of the column.

**Figure 29:**
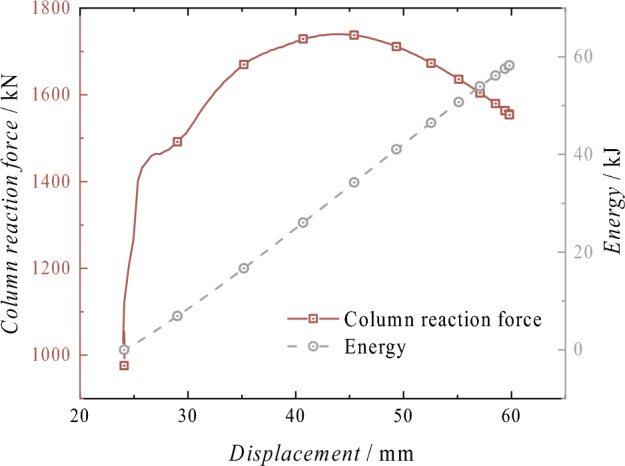
Reaction force–displacement and energy absorption curves of the energy absorption column.

**Figure 30:**
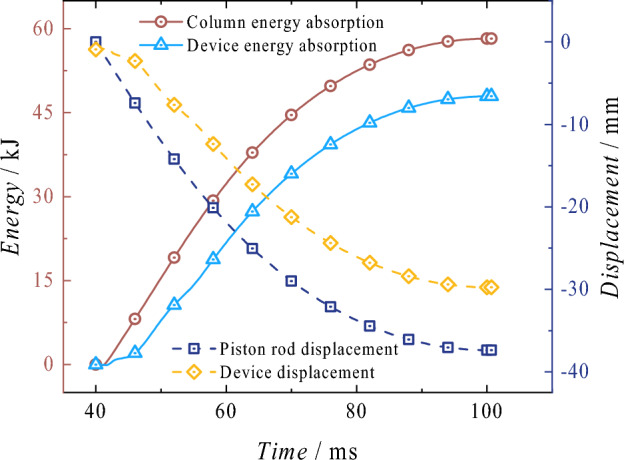
Energy-absorbing column and energy-absorbing device energy absorption process comparison diagram.

## Comparison and analysis of conventional columns and energy-absorbing columns

Under the same boundary conditions, a comparison is made between conventional columns and energy-absorbing columns, under the premise of a static load pressure of 1000 kN and an impact force of 1500kN (equivalent to a kinetic energy of 15456 J). The ratio of energy-absorbing columns to conventional columns is calculated. The results are shown in Table 4. Under the protection of the energy-absorbing device, the maximum reaction force of the energy-absorbing column is reduced by 32.52% compared to the conventional column. The maximum velocity of the piston rod and the maximum acceleration of the mass are significantly reduced by 24.08% and 59.46% respectively. The energy-absorbing column can provide a longer effective yielding displacement, enhancing it by 239.45% compared to the conventional column. This effectively delays the duration of the impact. In terms of energy absorption, the addition of energy-absorbing devices increases the absorption energy by 33.46%. Among them, the energy absorption of the conventional column itself is 43.63 kJ, while the energy absorption of the column itself in the energy-absorbing column is 10.29 kJ, which is only 23.58% of the energy absorbed by the conventional column. Most of the energy is absorbed by the energy-absorbing device. Due to the significant reduction in the support reaction force, the maximum stress and radial expansion of the cylinder are also reduced by 33.97% and 34.95% respectively. This reduction decreases the possibility of cylinder expansion. Comparing the rise and fall pressure rates of the entire impact process, it can be observed that the addition of the energy-absorbing device effectively slows down both the rise and fall pressure rates, with a decrease of 37.25% and 65.63% respectively.

**Table 4:** Comparison table of the dynamic parameters of conventional column and energy-absorbing column.

Parameter	Conventional column	Energy-absorbing column	Rate %
Reaction force/kN	2578.16	1739.7	↓32.52
Maximum velocity of column/(m/s)	1.91	1.45	↓24.08
Maximum acceleration of mass/mm/(ms^2^)	68.35	27.71	↓59.46
Displacement/mm	10.24	34.76	↑239.45
Duration of impact/ms	61.33	73.33	↑19.57
Impact process energy absorption/kJ	43.63	58.23	↑33.46
Maximum cylinder stress/MPa	379.48	250.56	↓33.97
Maximum cylinder radial expansion/mm	0.186	0.121	↓34.95
Rise pressure rate/(kN/ms)	59.94	37.61	↓37.25
Fall pressure rate/(kN/ms)	40.09	13.78	↓65.63

In summary, when dealing with impact loads, the energy-absorbing column can reduce the maximum impact force on the column, achieve a "peak shaving" effect, and effectively protect the column. It absorbs a significant amount of external impact energy, providing sufficient time for the safety valve to open. Compared to conventional columns, energy-absorbing columns demonstrate ideal advantages in various aspects.

## Conclusion

1. Based on the energy theory, a simplified equivalent model of conventional columns is established, considering the dynamic parameters when the safety valve fails to open effectively during a rock burst. The theoretical analytical expressions of various parameters are derived.
2. A conventional column CEL fluid–solid coupling model with a diameter of φ180 mm is established. The model is subjected to a static load pressure of 1000 kN and an impact pressure of 1500 kN. During the impact process, the stress state of the cylinder is significant, and significant peak points appear at the bottom of the cylinder and the end of the piston rod. The maximum surface stress is 379.48 MPa. The column absorbs 43.63 kJ of energy during the impact, which lasts for a total of 61.33 ms. The effective displacement is only 10.24 mm, and the radial expansion of the cylinder is 0.186 mm.
3. The 6500 kN static-dynamic combined hydraulic impact test machine is used for test verification. The results of the support reaction force, displacement, impact time, and cylinder strain are compared and analyzed with theoretical, experimental, and simulated data. The simulation error is below 25%, effectively confirming the accuracy and reliability of the CEL simulation algorithm.
4. An equivalent CEL model of the energy-absorbing column is established. Under the same boundary conditions, a comparison with the conventional column shows that the energy-absorbing column can effectively reduce the peak reaction force, stress, and radial expansion of the cylinder. It significantly improves energy absorption, slows down the acceleration of mass movement, effectively protects the column, increases displacement while delaying the impact duration, reduces the rise and fall pressure rate, and effectively controls mass movement while providing a time guarantee for the opening of the safety valve.

## Data Availability

The datasets generated and/or analysed during the current study are not publicly available due to our laboratory's current policies and confidentiality agreements regarding research on this subject but are available from the corresponding author on reasonable request.
